# Activity Recognition Using Hybrid Generative/Discriminative Models on Home Environments Using Binary Sensors

**DOI:** 10.3390/s130505460

**Published:** 2013-04-24

**Authors:** Fco. Javier Ordóñez, Paula de Toledo, Araceli Sanchis

**Affiliations:** Computer Science Department, University Carlos III of Madrid, Leganés, Madrid 28911, Spain; E-Mail: mtoledo@inf.uc3m.es (P.T.); masm@inf.uc3m.es (A.S.)

**Keywords:** activity recognition, hidden Markov model, hybrid schemes, wireless sensor networks

## Abstract

Activities of daily living are good indicators of elderly health status, and activity recognition in smart environments is a well-known problem that has been previously addressed by several studies. In this paper, we describe the use of two powerful machine learning schemes, ANN (Artificial Neural Network) and SVM (Support Vector Machines), within the framework of HMM (Hidden Markov Model) in order to tackle the task of activity recognition in a home setting. The output scores of the discriminative models, after processing, are used as observation probabilities of the hybrid approach. We evaluate our approach by comparing these hybrid models with other classical activity recognition methods using five real datasets. We show how the hybrid models achieve significantly better recognition performance, with significance level *p* < 0.05, proving that the hybrid approach is better suited for the addressed domain.

## 1. Introduction

Population aging is currently having a significant impact on health care systems [1]. Improvements in medical care are resulting in increased survival into old age, thus cognitive impairments and problems associated with aging will increase [2]. It has been estimated that one billion people will be over the age of 60 by the year 2025 [3]. As the burden of healthcare on society increases, the need for finding more effective ways of providing care and support to the disabled and elderly at home becomes more predominant. Automatic health monitoring systems are considered a key technology in this challenge [4], because they can serve a dual role: (1) to increase the safety and the sense of security of people living on their own; and (2) to allow elderly patients to be self-reliant longer, fostering their autonomy [5].

Monitoring human activities of daily living (ADL), in order to assess the cognitive and physical wellbeing of elderly, is considered a main aspect in building intelligent and pervasive environments [6]. Systems that recognize ADL from sensor data are now an active topic of research; indeed diverse approaches have been proposed to deal with the activity recognition problem, ranging from video cameras [7], RFID readers [8] and wearable sensors [9]. However, Wireless Sensor Networks (WSN) are considered one of the most promising technologies for enabling health monitoring at home due to their suitability to supply constant supervision, flexibility, low cost and rapid deployment [10,11]. Besides, the inherent non-intrusive characteristics of these networks have been proved to suit perfectly with environments where privacy and user acceptance is required [12]. Previous approaches have shown how simple binary sensors have solid potential for solving the ADL recognition problem in the home [13], and can be applied in human-centric problems such as health and elder care [14–16]. In Reference [17], binary sensors measuring the opening or closing of doors and cupboards, the use of electric appliances, as well as motion sensors were used to recognize ADLs of elderly people living on their own. Indeed, this kind of sensors is considered one of the most promising technologies to solve key problems in the ubiquitous computing domain, due to their suitability to supply constant supervision and their inherent non-intrusive characteristics.

In different studies, several models have been used to recognize ADL from sensor streams, such as Bayesian Networks [14], Conditional Random Field [18] or Evolving Classifiers [19]. However, recognizing human activities has to cope with several challenges: each human performs each activity differently, the length of the activities is usually unknown and sensor data are noisy. Nevertheless, temporal probabilistic models provide a good framework to handle the uncertainty caused by these issues. Specifically, the hidden Markov model (HMM) has been successfully applied in many sequential data modeling problems, and has been shown to perform well in this domain [8].

HMM can be effectively used for recognizing human activities, but modeling the emission probabilities when observable variables are defined by a collection of binary values can reach a high degree of complexity. To exactly model the distribution of the observation vector, all possible combinations of values in the feature space have to be considered, resulting in a large number of parameters and requiring accordingly large numbers of training elements. As demonstrated by Kasteren *et al.*, the most plausible solution to this problem is to use a naive Bayes assumption, meaning that strong model assumptions, as the complete independence of every feature, must be applied [20]. In this paper we postulate that the combination of the discriminative capabilities of a machine learning scheme, such as an artificial neural network (ANN) or a support vector machine (SVM), and the superior dynamic time warping abilities of HMM can offer better results for the dynamic pattern recognition task addressed in this domain.

The resulting model is denoted as a hybrid HMM approach, where the temporal characteristics of the data are modeled by HMM state transitions and a machine learning scheme is used to model HMM state distributions. An important advantage of such hybrid models is that existing methods for HMM design, training and recognition can be employed without significant modifications, since the hybrid HMM behaves essentially as a conventional HMM.

Different types of hybrid HMM systems have successfully been applied in diverse domains. A particularly popular approach is to combine HMMs with ANNs. Rynkiewicz applied a hybrid HMM/ANN scheme to predict time series data, obtaining a model that gave a much better segmentation of the series [21]. Models based on a hybrid ANN framework have been also widely used on various recognition tasks, namely: speech recognition [22,23], handwritten text recognition [24], sentence recognition [25] and digit recognition [26].

Other hybrid HMM systems are also present in the literature. Stadermann *et al.*, presented an acoustic model combining SVMs and HMMs that obtained an improvement of the word error rate compared with baseline acoustic models [27]. Ganapathiraju *et al.*, also employed an implementation of a hybrid SVM/HMM system for speech recognition, where the SVMs were trained on segment level data with one-state HMMs [28]. In Reference [29], Markov *et al.*, used Bayesian Networks as speech models to create a hybrid HMM/BN acoustic scheme that achieved better performance than the conventional HMM.

In the activity recognition domain, hybrid approaches have been also successfully employed. In our recent work [30], we showed that an ANN could be hybridized with HMMs to deal with the activity recognition problem in a home setting. Lester *et al.*, developed a hybrid model that combined a modified version of AdaBoost with HMMs, and demonstrated it to be quite effective for recognizing various human activities using wearable devices [31].

This paper proposes two new approaches to recognize ADLs from binary sensor streams based on hybrid HMM schemes (combined with either ANN or SVM). We evaluate and compare the activity recognition performance of these models on multiple fully annotated real world datasets: three well known datasets generated by Kasteren *et al.*, and two new datasets (“OrdonezA” and “OrdonezB”) that we introduce in this paper. This kind of approach has been previously applied for recognizing human activities using wearable devices but not in a wireless sensor network setting, to the best of our knowledge. In our experiments, hybrid models outperform other classical activity recognition methods, showing that the combination of generative and discriminative models can result in a significant increase in recognition performance.

This paper is organized as follows. Section 2 gives an overview of the type of data used in this study. Section 3 details the structure of the model employed in this work. Section 4 describes the experimental setting and experimental results obtained. Finally, Section 5 presents our conclusions and future work.

## 2. Binary Sensor Data

In this paper we have employed datasets generated by a set of simple state-change sensors installed in five different environments. Each dataset is composed by binary temporal data from a number of sensing nodes that monitored the ADLs performed in a home setting by a single inhabitant. Three of these datasets have been broadly employed in previous studies [32,33] and are publicly available for download from Reference [34].

The datasets were obtained using similar sensor systems in different houses. The layout of the different home settings differs strongly, as well as the sensors configuration. The type of sensors employed to monitor the users was chosen according to two main criteria: ease of installation and minimal intrusion. Sensors that need to be worn on the body may be considered intrusive by the user, and sensors that are easy to install can increase the acceptance of the system.

The WSNs deployed in our different home environments were focused to measure equivalent things: passive infrared sensors to detect motion in a specific area; reed switches for open/close states of doors and cupboards, and float sensors to measure the toilet being flushed. An overview of the datasets can be found in Table 1.

**Figure 1.**
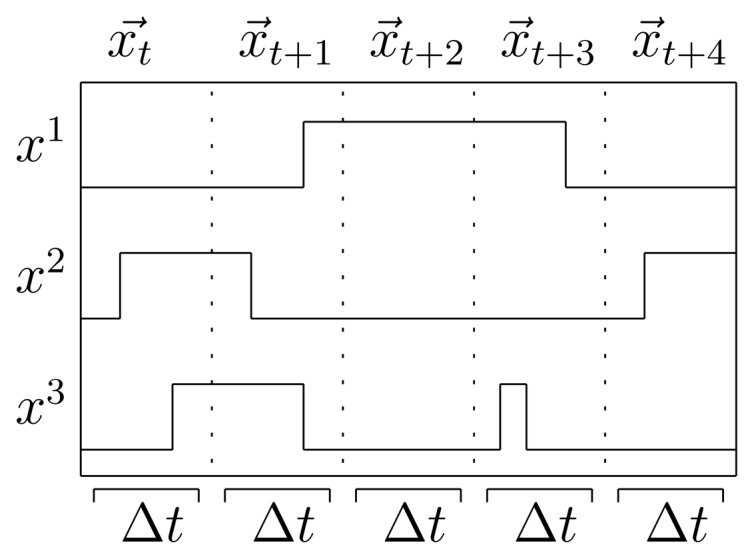
Temporal segmentation and relation between sensor readings *x^i^* and time intervals Δ*t*.

To provide a proper temporal format, the timeline is discretized into a set of time slices: measurements of the binary sensors taken at intervals that are regularly spaced with a predetermined time granularity Δ*t*. Sensor events for each time slice *t* are denoted as ${\text{x}}_{t}^{i}$, indicating whether sensor *i* fired at least once between time *t* and time *t* + Δ*t*, with ${\text{x}}_{t}^{i}\in \left\{0,1\right\}$. In a home setting with *N* state-change sensors, a binary observation vector ${\vec{\text{x}}}_{t}={\left({\text{x}}_{\text{y}}^{1},{\text{x}}_{t}^{2},\ldots ,{\text{x}}_{t}^{N-1},{\text{x}}_{t}^{N}\right)}^{T}$ is defined for each time slice. In the employed data representation, each time interval strictly corresponds to a single data instance. The class of each data instance is defined by the activity label of the corresponding time segment. The activity at time slice *t*, which is the state that the system is in, is denoted with *y_t_* ∈ {1,…, *Q*} for *Q* possible states, so the classification task is to find a mapping between a sequence of observations $\text{x}=\left\{\vec{{\text{x}}_{t1}},\vec{{\text{x}}_{t2}},\ldots ,\vec{{\text{x}}_{T}}\right\}$ and a sequence of labels **y** = {*y_t_*_1_, *yt_2_*, …, *y_t_*} for a total of *T* time intervals (see Figure 1).

## 3. The Hybrid HMM Approach

In our ADL recognition problem, the goal is to identify which activities took place given a sequence of sensor data. Therefore, we want to find the likeliest sequence of activities *y*_1:_*_T_* that best explains the sequence of observations *x*_1:_*_T_*. In a probabilistic framework, this problem corresponds to finding the sequence *y*_1:_*_T_* that maximizes the a posteriori probability *p*(*y*_1:_*_T_* |*x*_1:_*_T_*).

In this section, we describe the classic HMM, explain the probability distributions that make up such model and introduce the set of parameters underlying these distributions. Then, we present how to create the hybrid recognition system through the effective combination of the HMM with discriminative classifiers.

### 3.1. Hidden Markov Model

**Figure 2.**
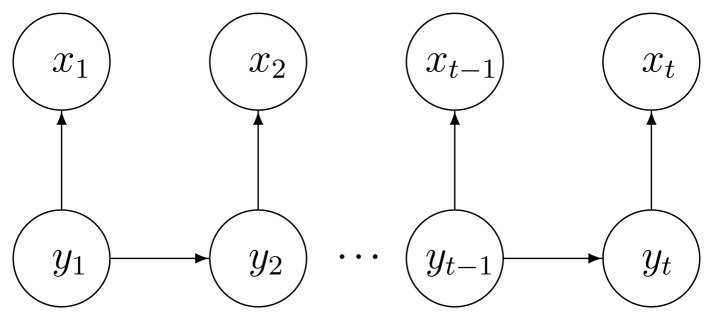
Graphical representation of HMM dependencies.

A standard HMM is a generative probabilistic model defined in terms of an observable variable x_t_ and a hidden variable y*_t_* at each discrete time instant. In our case the observable variable is composed by the features in the sensor feature space and the hidden variable is the ADL to recognize. Generative models provide an explicit representation of dependencies by specifying the factorization of the joint probability of the hidden and observable variables. The HMM is defined by two dependence assumptions, represented by the directed arrows in Figure 2.

- The hidden variable at time t, namely y*_t_*, depends only on the previous hidden variable y*_t_*_-1_ (first order Markov assumption [35]).
- The observable variable at time t, namely *x_t_*, depends only on the hidden variable y*_t_* at that time slice.

The joint probability therefore factorizes as follows:

(1) $$p({\text{y}}_{1:T},{\text{x}}_{1:T})=p({\text{y}}_{1})p({\text{x}}_{1}|{\text{y}}_{1})\prod_{t=2}^{T}p({\text{y}}_{t}|{\text{y}}_{t-1})p({\text{x}}_{t}|{\text{y}}_{t})$$

The different factors further specify the workings of the model. The initial state distribution *p*(y_1_) is a probability table with individual values denoted as follows:

(2) $$p({\text{y}}_{1}=i)\equiv \pi_{i}$$

The observation distribution *P*(x*_t_*|y*_t_*) indicates the probability that the state y*_t_* would generate observation x*_t_*. In our domain each binary sensor observation is modeled as an independent Bernoulli distribution, giving:

(3) $$p({\text{x}}_{t}|{\text{y}}_{t})=\prod_{n=1}^{N}p({\text{x}}_{t}^{n}|{\text{y}}_{t})$$

(4) $$p({\text{x}}_{t}^{n}=\upsilon |{\text{y}}_{t}=i)={\left(\mu_{in}\right)}^{\upsilon }{\left(1-\mu_{in}\right)}^{1-\upsilon }$$

The transition probability distribution p(y*_t_*|y*_t_*_-1_) represents the probability of going from one state to the next. This is given by a conditional probability table A, where individual transition probabilities are denoted as follows:

(5) $$p({\text{y}}_{t}=j|{\text{y}}_{t-1}=i)\equiv a_{ij}$$

Therefore, the entire model is fully specified by three probability distribution: the distribution over initial states parameterized by π{π*i*}; the transition distribution parameterized by A = {*a*_ij_}; and the observation distribution parameterized by B = {*μ_in_*}.

### 3.2. Hybrid Generative/Discriminative Modeling

**Figure 3.**
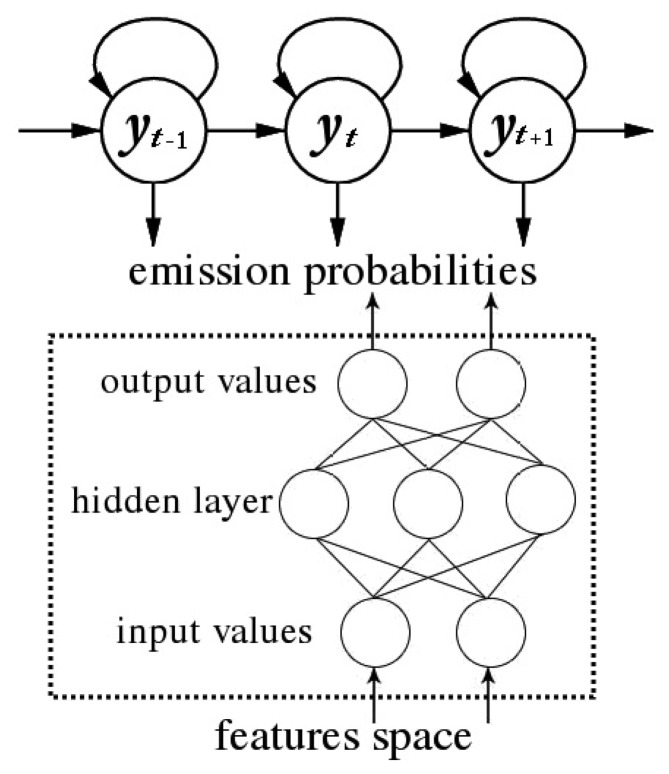
HMM/MLP model structure.

The hybrid HMM approach is a combination of an HMM that models the temporal characteristics of the sequential data and a static classifier that outputs a posterior probability for each label, taking as input the features in the sensor feature space. In this paper, we make use of two popular and powerful machine learning schemes as emission probability estimators: an ANN (Artificial Neural Network) and an SVM (Support Vector Machine). A standard HMM is employed to capture the temporal dynamics, but instead of directly using the sensor features to define an observation distribution, we trained the HMM employing the posterior probabilities obtained by the discriminative model selected (either an ANN or an SVM). A diagram of an example of hybrid HMM approach is shown in Figure 3.

The neural networks we used in this work are Multi-Layer Perceptrons (MLP) trained with the error back-propagation algorithm in order to maximize the relative entropy criterion. The support vector classifiers are widely used in diverse disciplines due to their high accuracy and ability to handle non-linear problems. In brief, these schemes apply a *kernel* function *K*(.) to the dot product of feature vector to avoid dealing directly with the high dimensional space and the excessive computations that result from such transformations [36]. From the choice of available kernel functions we have chosen the radial basis function (RBF) kernel *K*(*x*, *y*) = exp(−(|*x* − *y*|^2^)/(2σ^2^)), motivated by our experiments. Besides, in our case, the implementation of the SVM has to reduce our multiclass problem into multiple binary classification problems.

As previously explained, in a classic hidden Markov modeling approach, the emission probability density $p(\vec{{\text{x}}_{t}}|{\text{y}}_{t})$ has to be estimated for each state *y*_t_ of the Markov chain, that is, the probability of the observed sensor features x*_t_* given the hypothesized state y*_t_* of the model. However, since in the presented scheme the emission probabilities are provided with discriminative models, we took advantage of an important property of these models, which is that their outputs are estimates of posterior probabilities when trained for pattern classification.

MLP can be trained to approximate the posterior probabilities of states when each unit of the output layer is associated with a specific state of the model [37]. A common way to obtain such distribution for every state y ∈ {1,…, *Q*} is to use the softmax activation function at the output layer:

(6) $$p(\text{y}|\vec{\text{x}})=\frac{\text{exp}({\text{s}}_{\text{y}})}{\sum_{i\in Q}\text{exp}({\text{s}}_{i})}$$

where *s*_y_ is the y*th* output value before applying the softmax function.

Regarding the SVM, the transformation of the model's class distances to probabilities is done by applying a sigmoid function:

(7) $$p(\text{y}|\vec{\text{x}})=\frac{1}{1+A\exp(-s_{\text{y}}+B)}$$

where *s_y_* denotes the SVM output representing class y. The sigmoid function parameters *A* and *B* are estimated using the algorithm from Reference [38].

Hence, the output values of the classifiers are estimates of the probability distribution over states conditioned on the input:

(8) $$g_{{\text{y}}_{t}}(\vec{{\text{x}}_{t}})=p({\text{y}}_{t}|\vec{{\text{x}}_{t}})$$

with *g_yt_* denoting the output representing state yt. The a posteriori probability estimates from the output, $p({\text{y}}_{t}|\vec{{\text{x}}_{t}})$, can be transformed into emission probabilities required by HMMs, $p(\vec{{\text{x}}_{t}}|{\text{y}}_{t})$, by applying Bayes rule:

(9) $$p(\vec{{\text{x}}_{t}}|{\text{y}}_{t})=\frac{p({\text{y}}_{t}|\vec{{\text{x}}_{t}})p(\vec{{\text{x}}_{t}})}{p({\text{y}}_{t})}$$

The state priors *p*(*y_t_*) can be estimated from the relative frequencies of each state from the training data. Besides, the scaled likelihoods $p({\text{y}}_{t}|\vec{{\text{x}}_{t}})/p({\text{y}}_{t})$ can be directly used as emission probabilities in the addressed scheme since, during recognition, the scaling factor $p(\vec{{\text{x}}_{t}})$ is a constant for all states. Posterior probabilities are always forced to sum up to 1. Using this conversion, the discriminative models can be integrated into hybrid structural-connectionist models via a statistical framework [39], obtaining at each time slice the emission probabilities needed for the HMMs with better discriminating properties and without any hypothesis on the statistical distribution of the data.

Several studies have shown that incorporating the classification power and discriminating capabilities of such models with the temporal segmentation power and statistical modeling of HMMs results in a system that is better than either static classifiers or HMMs [40]. The benefits arising from using ANNs or SVMs as emission probability estimators are:

- They provide discriminant-based learning, suppressing incorrect classification.
- They do not need to treat features as independent. There is no need of any particular assumptions about the independence of input features and statistical distributions.
- They are robust against under-sampled training data, meaning that statistical pattern recognition can be achieved over an under-sampled pattern space.

On the other hand, one of the drawbacks of this hybrid approach is that we have to use fully labeled datasets to train the classifiers.

The hybrid HMM model training is done by an iterative Expectation-Maximization algorithm, as proposed by Reference [39]. The training procedure proceeds as follows:

1. Among the available labelled data, training and test subsets are chosen using the cross-validation mechanism.
2. Assign an initial nonzero value to transition probabilities of the HMM.
3. The training data are employed to train the corresponding classifier (either the MLP or the SVM).
4. Use the partially trained hybrid model to find the best state sequence applying the Viterbi algorithm. This Viterbi procedure uses the class priors estimated from the relative frequencies of each class in the training data.
5. The procedure is repeated until convergence.

## 4. Experimental Setup and Results

To properly evaluate the presented approach, it has been tested on a real domain using real datasets. In the experiments carried out, we compare the performance of the two hybrid approaches proposed with other well-known classifiers, and with a classic HMM, since such temporal probabilistic model has shown to perform well in this domain [32]. It should be noted that we have followed the recommendations from Brush *et al.* [41], in the experimentation process and in the presentation of the results for the activity recognition domain.

This section is organized as follows. We first give a description of the dataset and provide details of our experimental design. Then, we present the results and discuss the outcome.

### 4.1. Datasets

Five fully labeled datasets were employed to validate the proposed approach, generated using five different sensor networks. The activities or labels considered were not the same for every dataset. In the datasets generated by Kasteren et al. [34], eight different ADLs were included as labels, namely: “Leaving”, “Toileting”, “Showering”, “Sleeping”, “Breakfast”, “Dinner”, “Drink”. Time intervals with no corresponding activity are referred to as “Idle”. In the “Ordonez” datasets “Drink” labels are not present, nevertheless four additional activities are included, namely: “Lunch”, “Snack”, “Spare time”, “Grooming”. Table 2 shows the number of separate instances per activity in each dataset.

As previously mentioned, sensor data streams were divided in time slices of constant length. For these experiments, sensor data were segmented in intervals of length Δ*t* = 60 seconds, based on the contributions of Reference [20]. This interval length is considered long enough to be discriminative and short enough to provide good accuracy labelling results, since with larger time slices the shorter activities would not survive the discretization process. After segmentation, there were a total of 33,120 time slices for “KasterenA” dataset, 17,280 time slices for “KasterenB”, 24,480 time slices for “KasterenC”, 20,160 time slices for “OrdonezA” and 30,240 time slices for “OrdonezB” dataset.

### 4.2. Experimental Design

**Figure 4.**
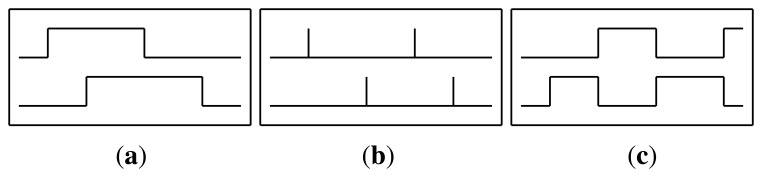
Considered feature representations. (**a**) Raw; (**b**) ChangePoint; (**c**) LastSensor.

The raw data streams generated by the sensor networks can either be used directly or preprocessed into a different representation form. In order to augment the features space and to obtain further evaluation of our models, in this work we have experimented with different feature representations, originally proposed by Kasteren *et al.* [32]. The sensor streams have been employed using three different representations:

- Raw: The raw sensor representation uses the sensor data in the same way it was received from the sensors network. The value is 1 when the sensor is active and 0 otherwise (see Figure 4(a)).
- ChangePoint: The change point representation indicates the moment when a binary sensor changes its value. That is, the value is 1 when a sensor state changes from zero to one or vice versa, and 0 otherwise (see Figure 4(b)).
- LastSensor: The last sensor representation indicates which sensor fired last. The sensor that changed state last continues to give 1 and only changes to 0 when another sensor changes its value (see Figure 4(c)).

During the experimentation these feature representations were used standalone and combined. Combining the feature representations was done by concatenating the feature matrices.

As can be noticed in Table 2, datasets suffer from a severe class imbalance problem due to the nature of the data. The class imbalance problem can be defined as a problem encountered by inductive learning systems on domains for which some classes are represented by a large number of examples while others are represented by only a few [42]. In learning extremely imbalanced data, the overall classification accuracy is considered not an appropriate measure of performance. A trivial classifier that predicted every instance as the majority class could achieve very high accuracy. Since in our case rare classes are of interest, we evaluate the models using F-Measure, a measure that considers the correct classification of each class equally important.

This measures can be calculated using the confusion matrix shown in Table 3. The diagonal of the matrix contains the true positives (*TP*), while the sum of a row gives us the total of true labels (*TT*) and the sum of a column gives us the total of inferred labels (*TI*). First, we calculate the precision and recall for each class separately and then take the average over all classes. F-Measure can be calculated from the precision and recall scores as follows:

(10) $$\text{F}‐\text{Measure}=\frac{2\cdot \text{precision}\cdot \text{recall}}{\text{precision}+\text{recall}}$$

The precision and recall metrics are defined as follows:

(11) $$\text{Precision}=\frac{1}{N}\sum_{i=1}^{N}\frac{TP_{i}}{TI_{i}}$$

(12) $$\text{Recall}=\frac{1}{N}\sum_{i=1}^{N}\frac{TP_{i}}{TT_{i}}$$

The models were validated splitting the original data into a test and training set using a “leave one day out” approach, retaining one full day of sensor readings for testing and using the remaining sub-samples as training data. The process is then repeated for each day and the average performance measure reported. Significance testing is done with significance level *p* < 0.05 using two different tests: a two-tailed Student t-test using matching paired data and a Wilcoxon signed-ranks test. We also perform a Wilcoxon significance test because Student t-test has shown a high probability of Type I errors when applied to repetitive random sampling or cross/leave-one-out validation [43].

### 4.3. Results

To evaluate the performance of the proposed hybrid approaches, they are compared with other classical activity recognition methods. As previously mentioned, two different discriminative models hybridized with HMMs are used in this work (HMM/MLP and HMM/SVM). Apart from a generative model (represented as a standard HMM), several well known classifiers are included in the comparison using a sliding window mechanism. It must be noted that, when using the HMM, each feature is modeled by an independent Bernoulli distribution, as proposed by previous studies [32].

The choice of the classifiers included in the comparison is based on the activity recognition study presented by Bao *et. al.* [44]. These discriminative models are: an MLP, an SVM, a tree-based classifier, a rule-based classifier and an instance-based classifier. Both MLP and SVM classifiers have the same configuration and topology as those hybridized with HMMs, which we have used to estimate the emission probabilities. For these classifiers, no function is applied to their output, since they are focused to directly recognize the activities, taking the sensor features as input. The tree-based classifier is modeled by the *C*4.5 algorithm [45], a widely employed algorithm to generate decision trees. The rule-based classifier is composed of propositional rules obtained through the Ripper algorithm [46]. The k-Nearest Neighbor (k-NN) algorithm [47] generates the instance-based classifier. The k-NN has to be parameterized with the number of neighbors (k) used for classification; in our case, our experiments showed that best results are obtained using *k* = 5.

Tables 4, 5, 6, 7 and 8 show the average F-Measure values for the five different datasets evaluated (“KasterenA”, “KasterenB”, “KasterenC”, “OrdonezA” and “OrdonezB” respectively). Rows correspond to the different feature representations employed (standalone and combined) and columns show the results of the experiments for each activity recognition model.

The results for datasets “KasterenA” and “KasterenB” are quite similar. With those datasets, the best results are obtained by the hybrid SVM/HMM approach, however the differences with some of the sliding window approaches (instance-based classifier, for example) are not statistically significant, with significance level p < 0.05. For dataset “KasterenC”, the hybrid MLP/HMM approach outperforms the other models, but the differences in this case cannot be considered to be significant either. Besides, with “KasterenB” and “KasterenC” datasets, both hybrid approaches significantly outperform the HMM approach.

Experiments carried out over the “OrdonezA” dataset show a clearly better F-measure performance for the hybrid schemes. In this case, the increase in performance for such hybrid approaches is statistically significant in all cases. On the other hand, although the HMM/SVM model outperforms the HMM hybridized with an MLP, the differences are not significant.

The results in the last test, over the “OrdonezB” dataset, are consistent with the experimentation data. The hybrid models achieve the best F-Measure value, but the difference with other models is considered to be not statistically significant in some cases.

Regarding the type of representation employed, in general terms, the best results are obtained using the “LastSensor” configuration as standalone and the “ChangePoint + LastSensor” concatenation as combined representation.

**Figure 5.**
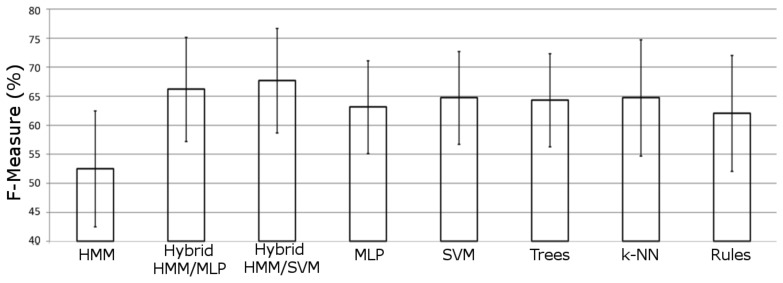
Averaged performance for each model in the comparison.

In Figure 5, the averaged performance over all datasets is shown for each model included in the experimentation. It can be noticed how both hybrid models significantly outperform the other approaches. The SVM hybrid model is the scheme that offers the best performance for this domain, significantly outperforming all other approaches. Both significance tests (Student t-test and Wilcoxon signed-ranks test) reveal how there are significant differences in the performance between hybrid MLP and hybrid SVM approaches.

This finding shows that combining the classification skills of a discriminative model with the generative and temporal powers of HMMs can lead to significantly better performance in real world activity recognition. In our experimentation, the generative/discriminative combination has been proved to outperform as much to the HMM as to the discriminative model employed (either the MLP or the SVM).

Besides, it is also remarkable that, when dealing with binary sensor features, the activity recognition algorithm based on SVM generalizes better than the MLP approach, in both hybrid and sliding window configurations. In some cases, differences in the results for the MLP hybrid approach are not even significant when compared with other models that used the sliding window mechanism (for instance when compared with the SVM). However, in both cases the recognition power of the discriminative models increases when they are combined with the ability of HMM to deal with temporal patterns.

## 5. Conclusions

In this paper we have proposed two new approaches to recognize ADLs from home environments using a network of binary sensors. Experimental results of the hybrid HMM models presented demonstrate how different hybrid schemes can be effectively employed for activity recognition in a home setting. Specifically, we show how the hybrid system obtained by using an SVM to estimate the emission probabilities of an HMM outperforms other well known sequential pattern recognition approaches. By incorporating the time modelling abilities of the HMM to the discriminative skills of the classifier we obtain an efficient scheme that is able to deal with the diverse statistical challenges presented in recognizing human activities and overcome the weakness of HMMs as effective classifiers. Considering the performance of the two hybrid approaches evaluated, the results show how the combination of discriminative and generative models is more accurate than either of the models on their own. Besides, when comparing the proposed hybrid approaches with other classifiers in terms of F-measure, hybrid schemes show a significantly better performance, with significance level *p* < 0.05, in both Student and Wilcoxon significance tests. It is also remarkable that the proposed hybrid models do not require to apply model assumptions and can estimate the emission probabilities with better discriminating properties, increasing the observations space and, without any hypotheses on the statistical distribution of the data, showing how the proposed system is a proper approach to deal with the addressed problem.

Among the different schemes evaluated, the SVM/HMM hybrid approach obtains a significant and notable better performance. We consider that SVM based approaches have great potential and further uses in this human activity recognition problem. However, it must be noticed that hybridizing these schemes implies a more complex system; hence, when integrating into a real home monitoring solution, it should be considered whether performance should take priority over efficiency. Fortunately, the training phase in a deployed activity recognizer is usually done offline, so we do not consider such growth of complexity a real problem in our domain.

Furthermore, the work presented here further demonstrates that accurate ADL recognition can be achieved by a set of simple and cheap state-change sensors installed in a wireless network.

In terms of future work, further extensions of the hybrid models are feasible, being possible to employ different classifiers as the discriminative layer of our approach. Also, due to the fact that the hybrid schemes can estimate the emission probabilities with better discriminating properties, it would be valuable to evaluate our approaches with non-binary sensor datasets.

## Figures and Tables

**Table 1.** Home settings description.

	**KasterenA**	**KasterenB**	**KasterenC**	**OrdonezA**	**OrdonezB**
Setting	Apartment	Apartment	House	House	House
Rooms	3	2	6	4	5
Duration	22 days	12 days	17 days	14 days	21 days
Sensors	14	23	21	12	12

**Table 2.** Percentage of instances per class for each dataset.

**Activity**	**KasterenA**	**KasterenB**	**KasterenC**	**OrdonezA**	**OrdonezB**
Leaving	49.74%	54.36%	46.27%	8.32%	17.41%
Toileting	0.65%	0.27%	0.62%	0.76%	0.55%
Showering	0.7%	0.6%	0.6%	0.54%	0.24%
Sleeping	33.42%	33.53%	28.46%	39.1%	35.58%
Breakfast	0.23%	0.52%	0.62%	0.63%	1.02%
Dinner	1.0%	0.42%	1.26%	0%	0.38%
Drink	0.1%	0.07	0.11%	0%	0%
Idle/Unlabeled	14.12%	10.12%	21.97%	5.61%	11.73%
Lunch	0%	0%	0%	1.59%	1.30%
Snack	0%	0%	0%	0.05%	1.33%
Spare time/TV	0%	0%	0%	42.7%	28.98%
Grooming	0%	0%	0%	0.73%	1.42%

**Table 3.** Confusion Matrix showing the true positives (*TP*), total of true labels (*TT*) and total of inferred labels (*TI*) for each class.

**True**	**Inferred**			

	**1**	**2**	**3**	
1	*TP*_1_	∊_12_	∊_13_	*TT*_1_
2	∊_21_	*TP*_2_	∊_23_	*TT*_2_
3	∊_31_	∊_32_	*TP*_3_	*TT*_3_

	*TI*_1_	*TI*_2_	*TI*_3_	*Total*

**Table 4.** Experimental results for dataset “KasterenA”. Average F-Measure (expressed in %).

**Dataset KasterenA**								

		**Hybrid Models**		**Schemes**				
			
**Representation**	**HMM**	**MLP**	**SVM**	**MLP**	**SVM**	**Trees** **(k=5)**	**k-NN**	**Rules**
Raw	41 ± 20	55 ± 12	58 ± 12	51 ± 11	54 ± 10	53 ± 12	55 ± 11	52 ± 12
ChangePoint	72 ± 14	56 ± 11	76 ± 9	50 ± 11	52 ± 11	54 ± 10	54 ± 10	54 ± 11
LastSensor	61 ± 15	60 ± 12	62 ± 12	61 ± 11	61 ± 11	59 ± 11	61 ± 11	61 ± 11
Raw&CP	51 ± 20	57 ± 11	65 ± 9	54 ± 10	56 ± 10	56 ± 12	58 ± 11	54 ± 13
Raw&LS	69 ± 13	69 ± 11	72 ± 9	67 ± 10	67 ± 8	69 ± 9	69 ± 7	65 ± 10
CP&LS	72 ± 15	71 ± 10	76 ± 8	68 ± 8	67 ± 8	68 ± 7	69 ± 7	67 ± 12
Raw&CP&LS	70 ± 14	68 ± 12	73 ± 9	68 ± 8	70 ± 8	69 ± 7	70 ± 7	63 ± 11

**Average**	62± 15	62± 11	69± 10	60± 9	61± 10	61± 10	62± 9	59± 11

**Table 5.** Experimental results for dataset “KasterenB”. Average F-Measure (expressed in %).

**Dataset KasterenB**								

		**Hybrid Models**		**Schemes**				
			
**Representation**	**HMM**	**MLP**	**SVM**	**MLP**	**SVM**	**Trees**	**k-NN** **(k=5)**	**Rules**
Raw	39 ± 13	53 ± 9	51 ± 10	50 ± 10	57 ± 10	51 ± 10	54 ± 11	48 ± 12
ChangePoint	51 ± 16	60 ± 9	73 ± 11	53 ± 5	56 ± 6	58 ± 7	58 ± 6	58 ± 8
LastSensor	40 ± 17	65 ± 9	63 ± 10	65 ± 12	65 ± 12	64 ± 12	65 ± 12	64 ± 12
Raw&CP	28 ± 10	54 ± 9	56 ± 14	53 ± 10	57 ± 8	55 ± 10	55 ± 7	51 ± 15
Raw&LS	37 ± 12	54 ± 15	60 ± 12	55 ± 11	60 ± 8	59 ± 9	63 ± 9	49 ± 12
CP&LS	44 ± 9	72 ± 11	72 ± 10	65 ± 8	63 ± 7	68 ± 7	66 ± 8	66 ± 9
Raw&CP&LS	42 ± 10	60 ± 11	65 ± 14	57 ± 9	63 ± 6	61 ± 8	65 ± 8	49 ± 10

**Average**	40± 12	60± 10	63± 12	57± 9	60± 8	60± 9	61± 9	55± 11

**Table 6.** Experimental results for dataset “KasterenC”. Average F-Measure (expressed in %).

**Dataset KasterenC**								

		**Hybrid Models**		**Schemes**				
			
**Representation**	**HMM**	**MLP**	**SVM**	**MLP**	**SVM**	**Trees** **(k=5)**	**k-NN**	**Rules**
Raw	15 ± 8	50 ± 12	45 ± 10	50 ± 10	49 ± 9	50 ± 8	48 ± 10	44 ± 11
ChangePoint	45 ± 8	59 ± 6	58 ± 10	47 ± 8	46 ± 9	45 ± 7	46 ± 7	45 ± 6
LastSensor	46 ± 12	66 ± 7	63 ± 6	67 ± 7	67 ± 7	66 ± 8	67 ± 7	67 ± 7
Raw&CP	46 ± 10	50 ± 9	49 ± 8	47 ± 7	51 ± 8	49 ± 8	47 ± 9	43 ± 14
Raw&LS	46 ± 11	58 ± 10	57 ± 8	57 ± 10	61 ± 6	62 ± 7	62 ± 8	48 ± 12
CP&LS	40 ± 16	66 ± 9	62 ± 7	62 ± 8	66 ± 8	65 ± 9	64 ± 8	65 ± 8
Raw&CP&LS	47 ± 12	61 ± 8	59 ± 8	61 ± 9	62 ± 7	65 ± 7	62 ± 8	51 ± 9

**Average**	40 ± 11	59 ± 9	56 ± 8	56 ± 8	57 ± 8	57 ± 8	57 ± 8	52 ± 10

**Table 7.** Experimental results for dataset “OrdonezA”. Average F-Measure (expressed in %).

**Dataset OrdonezA**								

		**Hybrid Models**		**Schemes**				
			
**Representation**	**HMM**	**MLP**	**SVM**	**MLP**	**SVM**	**Trees**	**k-NN** **(k=5)**	**Rules**
Raw	51 ± 7	78 ± 7	79 ± 5	77 ± 7	78 ± 7	78 ± 7	77 ± 5	78 ± 7
ChangePoint	57 ± 5	61 ± 7	64 ± 7	52 ± 7	53 ± 7	52 ± 7	53 ± 7	52 ± 7
LastSensor	54 ± 7	71 ± 7	67 ± 10	67 ± 8	66 ± 8	65 ± 8	65 ± 8	65 ± 8
Raw&CP	51 ± 5	81 ± 7	79 ± 6	77 ± 7	78 ± 7	78 ± 7	76 ± 7	78 ± 7
Raw&LS	56 ± 5	82 ± 5	83 ± 8	82 ± 7	84 ± 7	82 ± 7	80 ± 86	83 ± 8
CP&LS	50 ± 7	72 ± 7	72 ± 10	72 ± 8	71 ± 8	72 ± 7	71 ± 8	69 ± 8
Raw&CP&LS	53 ± 5	82 ± 5	83 ± 7	82 ± 7	84 ± 7	83 ± 7	79 ± 8	83 ± 7

**Average**	53± 05	75± 6	76± 7	73± 7	73± 7	73± 7	72± 18	72± 7

**Table 8.** Experimental results for dataset “OrdonezB”. Average F-Measure (expressed in %).

**Dataset OrdonezB**								

		**Hybrid Models**		**Schemes**				
			
**Representation**	**HMM**	**MLP**	**SVM**	**MLP**	**SVM**	**Trees**	**k-NN** **(k=5)**	**Rules**
Raw	69 ± 7	74 ± 6	74 ± 8	68 ± 6	69 ± 7	69 ± 7	69 ± 7	68 ± 6
ChangePoint	65 ± 8	61 ± 12	68 ± 6	50 ± 7	50 ± 5	51 ± 7	52 ± 7	51 ± 6
LastSensor	62 ± 6	72 ± 6	70 ± 7	72 ± 7	73 ± 7	71 ± 7	73 ± 7	73 ± 7
Raw&CP	69 ± 7	75 ± 7	75 ± 6	68 ± 7	69 ± 7	69 ± 8	69 ± 8	69 ± 6
Raw&LS	67 ± 6	71 ± 7	74 ± 6	74 ± 7	76 ± 6	72 ± 6	76 ± 7	76 ± 6
CP&LS	66 ± 6	70 ± 7	72 ± 7	71 ± 8	74 ± 7	71 ± 6	73 ± 7	72 ± 6
Raw&CP&LS	66 ± 7	72 ± 7	74 ± 6	73 ± 7	76 ± 7	74 ± 6	76 ± 8	77 ± 6

**Average**	66± 06	71± 7	72± 7	68± 7	69± 7	68± 7	70± 7	69± 6
